# Evaluation of the SaCoVLM, a novel third-generation video laryngeal mask: a prospective observational pilot study

**DOI:** 10.1186/s12871-026-03653-x

**Published:** 2026-01-28

**Authors:** Michal Kalina, Jan Beneš, Michal Hanauer, Jan Rejholec, Jakub Jobánek, Vladimír Černý

**Affiliations:** 1 Department of Anaesthesiology, Resuscitation and Intensive Care, Děčín Hospital, U nemocnice 1, Děčín, 40502 Czech Republic; 2 Department of Anaesthesiology, Perioperative Medicine and Intensive Care, J. E. Purkinje University, Masaryk Hospital in Usti nad Labem, Socialni Pece 3316/12A, Usti nad Labem, 40113 Czech Republic; 3 Faculty of Medicine in Hradec Kralove, Charles University, Simkova 870, Hradec Kralove, 50003 Czech Republic; 4 Department of Anesthesiology and Intensive Care Medicine, University Hospital Hradec Kralove, Sokolská 581, Hradec Kralove, 50005 Czech Republic; 5 Surgical Department, Děčín Hospital, U nemocnice 1, Děčín, 40502 Czech Republic; 6 Department of Anaesthesia, Pain Management and Perioperative Medicine, Dalhousie University, Halifax, NS B3H4R2 Canada; 7 Department of Anaesthesia and Intensive Care Medicine, 3rd Faculty of Medicine Prague, Charles University, Ruska 97, Prague, 10000 Czech Republic; 8 Department of Anaesthesiology and Critical Care, Bulovka University Hospital, Budínova 67/2Libeň, , Praha 8, Libeň, 18081 Czech Republic

**Keywords:** Airway, Laryngeal mask, General anaesthesia, Adverse effects, Airway management, Patient safety

## Abstract

**Background:**

The laryngeal mask airway ( LMA ) is a well-established device for securing the airway, typically inserted using a blind technique. However, blind insertion may result in suboptimal alignment and the need for reinsertion, which can increase the risk of adverse events. Therefore, in this pilot study, we aimed to conduct a preliminary evaluation and gather initial clinical experience with the new video laryngeal mask, SaCoVLM™.

**Methods and materials:**

In this prospective observational study, the SaCoVLM™ was used for airway management in patients undergoing general anaesthesia. We assessed key quality parameters commonly used to compare the performance of different laryngeal masks. Primary outcome was first attempt success rate. Secondary outcomes were time to achieve adequate ventilation, oropharyngeal leak pressure (OLP), and the incidence of adverse events.

**Results:**

A total of 50 patients undergoing general anaesthesia were enrolled. The first-attempt insertion success rate was 96%. The mean time to achieve adequate ventilation was 13± 2.4 s, and the mean OLP was 38± 3.8 mbar. The overall incidence of adverse events related to video laryngeal mask usage was 6%, with no serious adverse events observed.

**Conclusion:**

The SaCoVLM™ appears to be a promising alternative for airway management in patients undergoing general anaesthesia, demonstrating favourable performance across key clinical parameters.

**Trial registration:**

The study was registered in the clinicaltrials.gov on 28th of May 2025 with study registration number NCT07007403 accessible on https://clinicaltrials.gov/study/NCT07007403.

## Background

The laryngeal mask (LMA) is a well-established device for securing the airway. Traditionally, it is inserted using a blind technique, as first described in the late 1980s. However, recent literature suggests that blind insertion may result in suboptimal mask alignment in up to 60% of cases, with up to 15% requiring reinsertion to achieve adequate ventilation [1–5]. The need for repositioning or reinsertion increases the risk of adverse events, including desaturation, hypoxia, laryngospasm, hoarseness, and soft tissue trauma [6].

These challenges may be overcome by video laryngeal masks (VLMA). The technology represents a promising advancement in supraglottic airway management. However, current devices remain limited in clinical availability and adoption [7]. Some VLMAs rely on external or detachable video components, which can complicate setup, limit portability, and increase costs [7]. Additionally, not all VLMAs provide a consistently clear or reliable view of the glottic structures, especially in the presence of secretions or blood [8]. Moreover, comprehensive clinical data comparing VLMAs to traditional LMAs in terms of safety, ease of use, and long-term outcomes are still limited, highlighting the need for further research and refinement of these devices [9].

The new video laryngeal mask airway (VLMA) SaCoVLM™ (Zhejiang UE Medical Corp., Zhejiang, China) incorporates a video system consisting of a video stylet and a dedicated video port, enabling real-time visualization of the glottis and surrounding anatomical structures during and after insertion. Unlike widely used laryngeal masks such as the LMA Supreme™, i-gel™, or LMA ProSeal™, which rely solely on blind placement, the SaCoVLM™ allows for direct visual confirmation of proper positioning. This feature represents a theoretical innovation, as it may offer the potential to assist with insertion guidance, facilitate early recognition of malposition, and enable adjustment during placement. Whether these features translate into improvements in ventilation or patient safety remains to be determined.

Therefore, we conducted this pilot study to evaluate the feasibility, safety, and performance of the SaCoVLM™ video laryngeal mask in a clinical setting. Since the available data on this new VLMA are limited, especially regarding the first insertion attempt success rate, time to adequate ventilation, sealing properties, and the incidence of adverse events, a preliminary investigation was necessary. The aim of this study was to gather initial clinical experience, identify potential complications, and assess key performance parameters prior to conducting a larger-scale prospective randomized trial directly comparing traditional laryngeal masks with video laryngeal masks. The primary outcome was the success rate of insertion on the first attempt, while secondary outcomes included oropharyngeal leak pressure, time to achieve adequate ventilation, and the incidence of adverse events.

## Methods

We conducted a single-center prospective observational study in patients undergoing general anaesthesia. The study was approved by the Ethics Committee of Krajská Zdravotní, a.s. - Děčín Hospital, Czech Republic (reference number 03/24). The study was done in accordance with the Ethical Principles for Medical Research Involving Human Subjects, outlined in the Helsinki Declaration of 1975 (revised 2013) (available from: https://www.wma.net/policies-post/wma-declaration-of-helsinki-ethical-principles-for-medical-research-involving-human-subjects/). The study was registered on the clinicaltrials.gov on the 28th of May 2025 with study registration number NCT07007403 accessible at https://clinicaltrials.gov/study/NCT07007403. Patients were enrolled from the 14th of June 2025 to the 30th of July 2025. Inclusion criteria were MACOCHA score ≤ 2, age ≥ 18 years. Exclusion criteria were age < 18 years, MACOCHA score > 2, other airway management preferable, refusal or failure to sign the informed consent for study participation, emergency cases, BMI > 35, ASA > II [10]. It was determined that 50 patients would be enrolled, based on the paper published by Sim [11].

### Objectives

The primary objective was to evaluate the first attempt success rate. Secondary objectives were the time needed to achieve adequate ventilation, OLP, and the incidence of adverse events.

### Study design

First, informed consent was obtained from the patient for participation in the study. The consent was obtained during the preoperative patient evaluation by the anaesthesiologist who provided anaesthesia care during the surgical procedure, in the preoperative holding area.

After obtaining informed consent, a standard preliminary examination was performed by the anaesthesiologist, along with routine preoperative preparation (e.g., establishment of peripheral venous access, airway risk assessment, etc.). The patient was subsequently transported to the operating room.

**Fig. 1:**
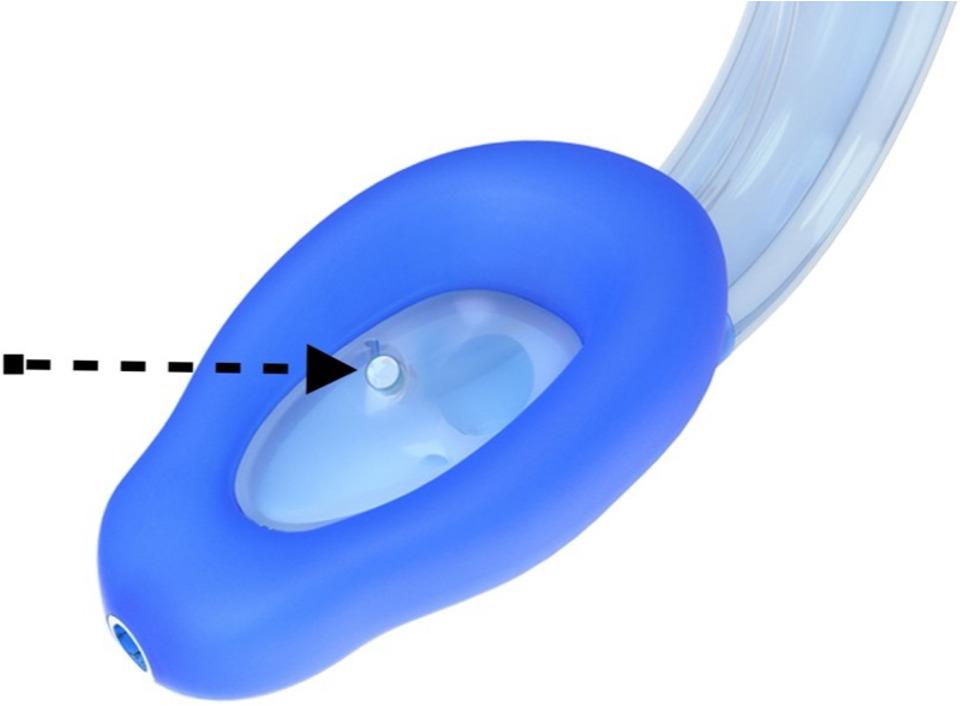
SaCoVLMTM the new third generation video laryngeal mask with the new design of obturation cuffs. Arrow pointing towards sealed vision window for video stylet. Image provided by medisap s.r.o. and reproduced with permission

**Fig. 2:**
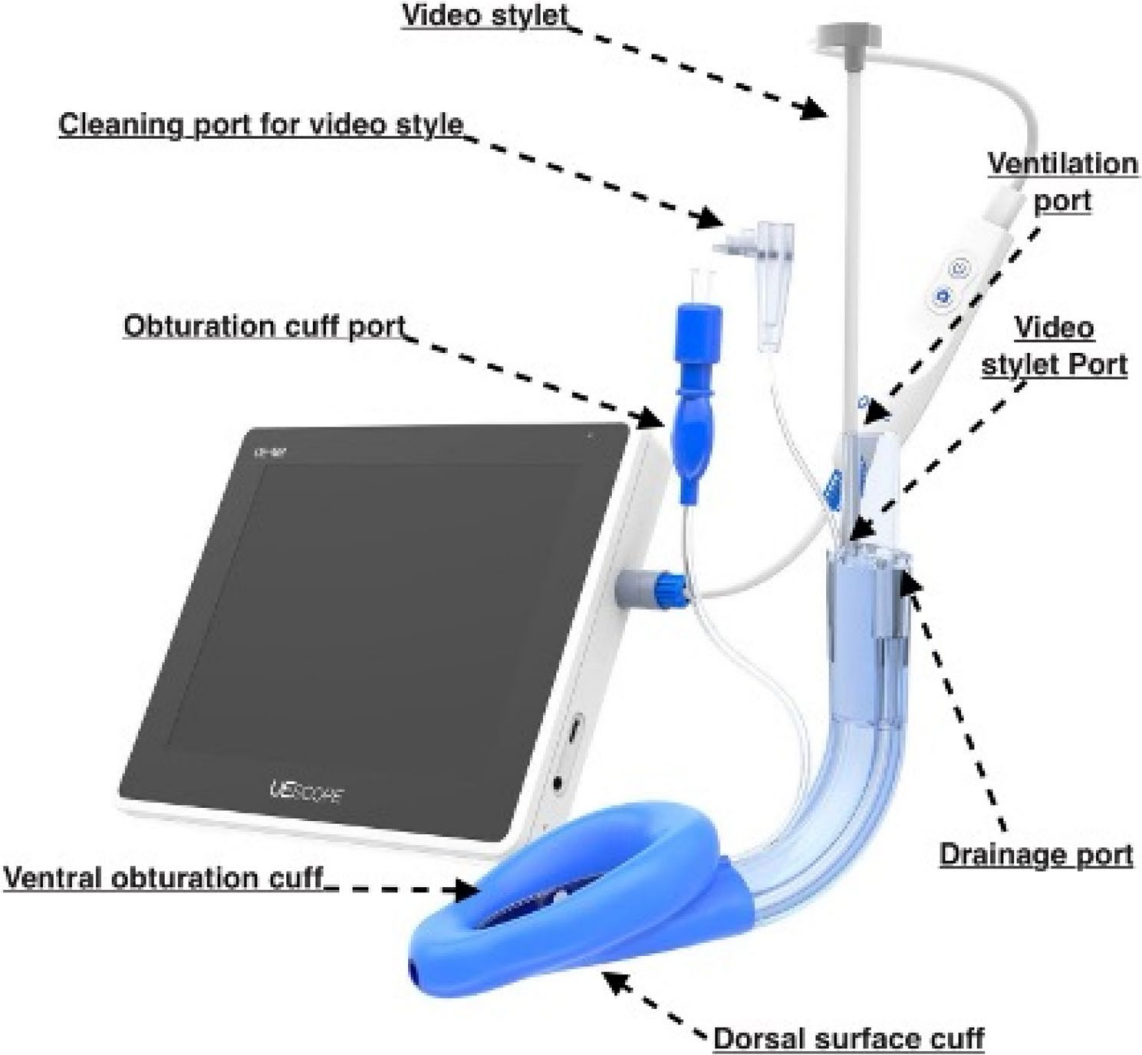
Detailed presentation of SaCoVLMTM set the new third generation video laryngeal mask with display. Image provided by medisap s.r.o. and reproduced with permission

**Fig. 3:**
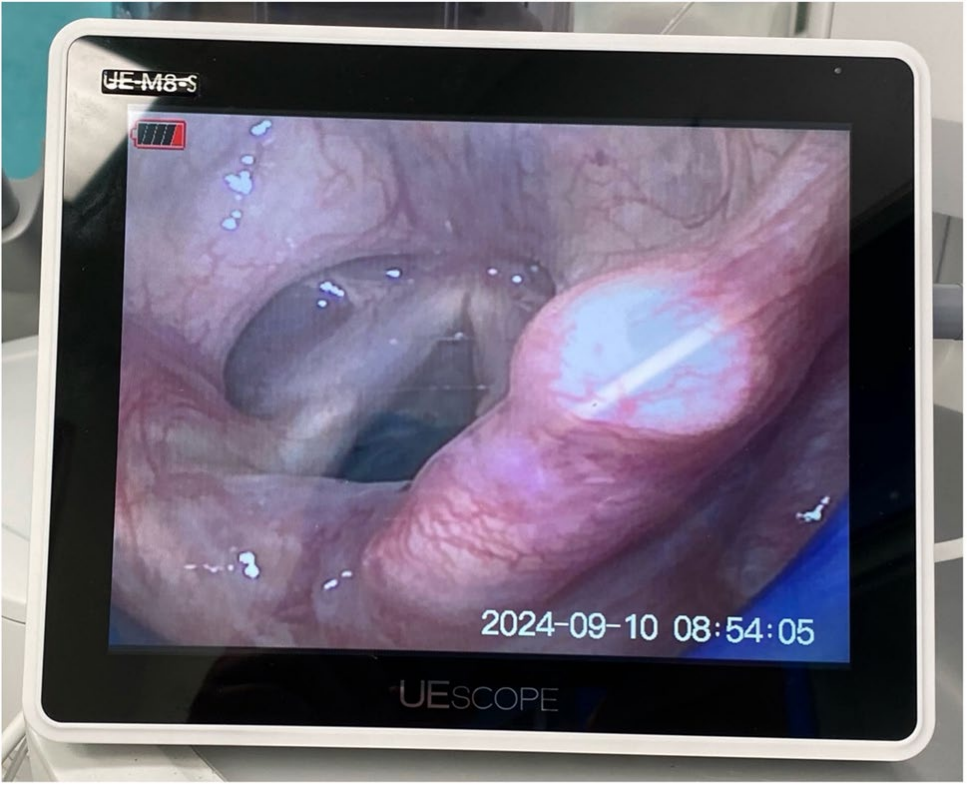
Visualization of the epiglottis and surrounding anatomical structures demonstrating correct positioning of the Video Laryngeal Mask Airway (VLMA)

Upon arrival in the operating room, standard patient monitoring was initiated in accordance with safe anaesthesia practice guidelines [12]. Anaesthesia induction followed. Anaesthesia induction was performed using propofol and sufentanil, with dosages determined at the discretion of the attending anaesthesiologist. Preoxygenation was performed with 100% oxygen at 8 L/min using a facemask in a 30^O^ head up position. No muscle relaxants were used to facilitate LMA insertion. Once adequate sedation depth was achieved—assessed by entropy (40 < SE < 60) — airway management was performed using the SaCoVLM™. The back of the mask was lubricated with a water-based gel. The anaesthetist inserted the video stylet into the laryngeal mask via dedicated video port and inserted the device under direct video guidance. In cases of obscured view due to mucus, the vision window was cleared using 20 ml of air via the designated cleaning port. The video laryngeal mask is presented in detail in Figs. 1 and 2. After placement of the laryngeal mask, the cuff was inflated using a manual manometer to achieve a pressure between 40 and 60 mbar. Visualization of the epiglottis and surrounding anatomical structures confirming correct positioning of the video laryngeal mask airway (VLMA) is shown in Fig. 3. After inflating the mask and checking of the correct position on the screen, mechanical ventilation was initiated.

After adequate ventilation was achieved, a gastric tube size of 12Fr was inserted through the drainage port with the aim of confirming the position of the drainage port over oesophagus. After 10 and 20 min from achieving the adequate ventilation, OLP was measured using airway pressure leak equilibrium according to Keller [13]. The surgical procedure then proceeded, during which the OLP was measured ten and twenty minutes from the incision. Ventilation during the surgery was maintained via the inserted laryngeal mask.

After removing the laryngeal mask at the end of the procedure, it was then documented whether there was any blood-stained secretion present on the LMA. The patient was thereafter transported to post anaesthesia care unit.

One hour after LMA removal, we recorded whether the patient experienced oropharyngeal discomfort (sore throat, pain, coughing, or haemoptysis). Additionally, the need for oxygen therapy was assessed at 10 min and at 1 h after LMA removal. Following this, the patient’s participation in the study ended and the patient was transferred to a ward.

### Measures

#### Success of the first attempt

We measured the success rate of the first insertion attempt. We defined the successful attempt as insertion of the LMA with adequate ventilation immediately after insertion.

#### Time needed to adequate ventilation

The time needed to achieve adequate ventilation was measured from the initiation of the insertion attempt until three plateau reading on the capnography.

#### Oropharyngeal pressure

We measured the OLP. The OLP is a crucial indicator of airway protection and the ability to deliver adequate positive pressure ventilation [14, 15]. The OLP was measured after the successful insertion of the LMA and confirmation of adequate ventilation, and after 10 and 20 min after achieving adequate ventilation. The mean value was calculated and used for further statistical evaluation. The fresh gas flow was set to 5 L per minute. Subsequently, the adjustable pressure-limiting (APL) valve was closed to its maximum setting (70 cm H₂O) and ventilation was switched to manual. Airway pressure was continuously monitored until reaching stability. The airway pressure stabilized after reaching an equilibrium with the leak. This measured pressure was considered the OLP [13].

#### Total number of attempts needed for successful insertion

The total number of insertion attempts was recorded, starting from the first attempt and continuing until successful VLMA placement with adequate ventilation was achieved.

#### SpO₂ at the time of ventilation initiation

The SpO2 value measured after achieving adequate ventilation. The value was recorded subsequent three plateau readings on capnography after successful insertion.

#### End tidal carbon dioxide after adequate ventilation initiation

The end tidal carbon dioxide level was measured after reaching adequate ventilation. We recorded the value from the third capnography reading.

#### Adverse events

We monitored adverse events during airway management with the SaCoVLM™ and during the postoperative period, up to one hour after LMA removal. Adverse events were defined as blood stains on the device, sore throat, regurgitation of gastric contents into the larynx, displacement of the LMA during the procedure, hoarseness, dysphagia, desaturation, and hypoventilation occurring during LMA insertion or the procedure itself. Desaturation and hypoventilation were considered adverse events when caused by LMA-related factors, such as malposition or displacement of the device.

### Statistical analysis

The mean values ± standard deviation (SD) or percentages were calculated as necessary. The normality of the data was evaluated according to the Kolmogorov- Smirnov test, confirming that the data were normally distributed. All calculations were performed in an open-source R environment using the ggplot2 library (v4.1.2, R Core Team (2021), R: A language and environment for statistical computing. R Foundation for Statistical Computing, Vienna, Austria, https://www.R-project.org/).

## Results

A total of 50 patients were enrolled in the study. Detailed study population demographics are presented in Table 1.

**Table 1:** Study population basic demography and characteristics of the studied population

Total number of participants (*n*)	50
Gender	
Male (*n*)	19 (38%)
Female (*n*)	31 (62%)
Age (years, mean ± SD)	56.3 ± 15.6
Height (cm, mean ± SD)	168.8 ± 11.7
Weight (kg, mean ± SD)	82.6 ± 11.7
BMI (kg/m^2^, mean ± SD)	28.6 ± 6.2
ASA	
I (*n*)	6
II (*n*)	44
Type of operation	
Gynaecological surgery (*n*)	6
Orthopaedic surgery (*n*)	4
Abdominal surgery (*n*)	36
Other type of surgery (*n*)	4
Capnoperitoneum	
Yes (*n*)	4
No (*n*)	46

We observed successful first insertion attempts in 48 cases, making the first insertion attempt success rate 96%. The mean time needed for achieving adequate ventilation was 13 s. The mean OLP we observed was 38 mbar. Results for primary and secondary outcomes are presented in Table 2. There were two cases in which reinsertion was needed. In both cases, a different size of the mask was used for the second insertion attempt. The mean SpO2 on ventilation start was 97 ± 2%, with a mean end tidal CO2 of 5.2 ± 0.7 kPa. In two cases, we observed blood stains on the dorsal part of the laryngeal mask after extraction, and in one case, sore throat was present without dysphagia or hoarseness. Apart from these, no adverse events, including hypoxia and hypoventilation, were observed during the study.

**Table 2:** Results for primary and secondary outcomes

Successful first insertion attempt (*n*)	48
Time to adequate ventilation (s, mean ± SD)	13± 2.4
Oropharyngeal leak pressure (mbar, mean ± SD)	38± 3.8
Adverse events (n)	3

## Discussion

Video guidance during LMA insertion allows operators to visualize the glottis and surrounding structures in real time, enabling immediate adjustments to ensure optimal placement. This direct feedback may reduce malposition rates and the need for subsequent repositioning compared to a blind technique. For example, fibreoptic evaluations of blind-inserted LMAs revealed suboptimal positioning in 40–60% of cases, often necessitating adjustment or reinsertion to restore adequate ventilation [2, 4, 5, 16]. Recent innovations, like the VLMA SaCoVLM™, are promising. Therefore, we evaluated the performance of the new VLMA SaCoVLM™ across several key quality indicators, including first-attempt insertion success, time to achieve adequate ventilation, OLP, and the incidence and severity of adverse events.

A successful first insertion attempt was observed in 48 out of 50 cases, resulting in a success rate of 96%. When compared to other third-generation laryngeal masks such as the Supreme™ or i-Gel™, which report first-attempt success rates ranging from 85% to 96%, the SaCoVLM™ demonstrates a comparable level of performance in terms of successful first insertion attempt [17–20].

We observed a shorter insertion time for the SaCoVLM™ compared to the Supreme™ and ProSeal™ laryngeal masks, as reported in the literature. The new video laryngeal mask was inserted in less than 14 s, whereas reported insertion times for the Supreme™ and ProSeal™ are approximately 20 s and 30 s, respectively [20]. We explain the shorter insertion time of the SaCoVLM™ by its improved curved design, which facilitates smoother anatomical guidance during placement.

Another key quality indicator of laryngeal mask performance is the OLP. This parameter reflects the quality of the airway seal, the effectiveness and safety of positive pressure ventilation, and helps predict the suitability of the device in various clinical scenarios. A higher OLP implies a superior seal, which is critical in positive pressure ventilation or in procedures requiring Trendelenburg positioning [21]. Additionally, OLP serves as an indicator of proper placement and alignment of the laryngeal mask [21–25]. In our study, the SaCoVLM™ demonstrated a mean OLP of 38 mbar. To the best of our knowledge, only one other study has specifically investigated the OLP of the SaCoVLM™, and its findings are consistent with ours [1]. Compared to other laryngeal mask airways, the SaCoVLM™ exhibits a significantly higher OLP. When compared with devices such as the Supreme™, i-gel™, and ProSeal™, the SaCoVLM™ showed an OLP that was approximately 10 to 15 mbar higher, indicating a superior airway seal [23, 24, 26]. We explain this finding by the innovative design of the SaCoVLM™, particularly the shape of the obturation cuff and the presence of a second dorsal obturation cuff, which likely contribute to an improved alignment and thus improved oropharyngeal seal. Another factor that may explain the higher OLP, compared to other laryngeal masks, is the ability to use a video stylet, which allows for real-time visualization and adjustment of the device’s position, resulting in more accurate placement and alignment of the mask.

In our study, we observed only a few adverse events. Specifically, blood stains on the LMA were noted in two cases, and one patient reported a sore throat. No other adverse events were recorded. Notably, we did not observe any displacement of the laryngeal mask, even in cases where muscle relaxants were used or the patient’s position was changed during the procedure. In conclusion, the overall incidence of adverse events was 6%, which is comparable to that reported for iGel™ and lower than that for Supreme™ [19, 27]. Interestingly, Yan reported a sore throat in 13% of caes and blood stains in 7% when sing the SaCoVLM™ [1]. We explain the discrepancy between Yan’s findings and ours primarily by differences in insertion technique. In Yan’s study, the overall incidence of adverse events—particularly sore throat and bleeding—was also significantly higher than what is generally reported in patients in whom the LMA Supreme™ was used [19, 27]. Another possible contributing factor could be differences in the patient population, particularly ethnicity. Yan’s study was conducted in a hospital in China, whereas our study was performed in a hospital in Central Europe.

We acknowledge several limitations of this study. First, no comparator laryngeal mask airway was included, resulting in the absence of a control group and precluding any conclusions regarding superiority or an improved safety profile of the investigated device. This was a deliberate decision based on the pilot nature of the study, which primarily aimed to obtain initial clinical experience with the SaCoVLM™. Second, the SaCoVLM™ was evaluated exclusively in patients without predicted difficult airways. Owing to the limited available evidence regarding its performance in difficult airway scenarios, this approach was adopted to ensure patient safety by initially assessing the device in a lower-risk population. Although the present study was limited to elective low-risk patients, future trials should investigate the performance of the SaCoVLM™ in obese patients, emergency settings, and difficult airway populations. In addition, blinding was not incorporated into the study protocol, and only descriptive statistical methods were applied, as the primary objective of this pilot study was to assess feasibility and to collect initial key quality parameters of the video laryngeal mask airway prior to a prospective randomized trial. Finally, the number of enrolled patients was relatively small; however, this reflects the exploratory design of the study. The limited sample size was considered appropriate to assess feasibility, gather preliminary safety and performance data, and identify potential methodological challenges, rather than to draw definitive conclusions.

Future trial is designed as a prospective, multicentre randomized controlled trial comparing the video laryngeal mask SaCoVLM™ with the established second-generation laryngeal mask Supreme™. The hypothesis is that video laryngeal mask SaCoVLM™ provides higher oropharyngeal leak pressure compared with the second-generation laryngeal mask Supreme™. The primary outcome is oropharyngeal leak pressure. Secondary outcomes include the incidence of inadequate ventilation during surgery, time from the first insertion attempt to achievement of adequate ventilation, first-attempt success rate, and the incidence of adverse events. The trial is registered in the Jan Evangelista Purkinje University study registry (ID 19052025) and on ClinicalTrials.gov. The anticipated start of the trial is 1st of April 2026.

## Conclusion

The SaCoVLM™ demonstrated favourable performance metrics in elective surgical patients under general anaesthesia, with high first-attempt success, short insertion time, and excellent OLP. These preliminary findings support its further evaluation in larger, comparative studies and in more diverse clinical populations.

## Data Availability

Data are available at the corresponding author on reasonable request.

## Abbreviations

APL: Adjustable pressure limiting valve
LMA: Laryngeal mask
OLP: Oropharyngeal leak pressure
VLMA: Video laryngeal mask

## References

[1] Yan Cling, Chen Y, Sun P, Qv Z, yang, Zuo M. zhang. Preliminary evaluation of SaCoVLM™ video laryngeal mask airway in airway management for general anesthesia. BMC Anesthesiol. 2022;22(1):3. 34979936 10.1186/s12871-021-01541-0 PMC8722220

[2] Chandan SN, Sharma SM, Raveendra US, Rajendra Prasad B. Fiberoptic assessment of laryngeal mask airway placement: a comparison of blind insertion and insertion with the use of a laryngoscope. J Maxillofac Oral Surg. 2009;8(2):95–8. 23139483 10.1007/s12663-009-0025-8 PMC3453942

[3] Campbell RL, Biddle C, Assudmi N, Campbell JR, Hotchkiss M. Fiberoptic assessment of laryngeal mask airway placement: blind insertion versus direct visual epiglottoscopy. J Oral Maxillofac Surg. 2004;62(9):1108–13. 15346362 10.1016/j.joms.2003.10.014

[4] Behera B, Misra S, Bellapukonda S, Sahoo A. Impact of visually guided versus blind techniques of insertion on the incidence of malposition of Ambu ^®^ auragaintm in paediatric patients undergoing day care surgeries: A prospective, randomised trial. Indian J Anaesth. 2020;64(11):937. 33487677 10.4103/ija.IJA_557_20 PMC7814999

[5] Payne J. The use of the fibreoptic laryngoscope to confirm the position of the laryngeal mask. Anaesthesia. 1989;44(10):865–865.2521122

[6] Brimacombe JR. Problems with the Laryngeal Mask Airway: Prevention and Management. Int Anesthesiol Clin. 1998;36(2):139-154. Available from: https://journals.lww.com/anesthesiaclinics/fulltext/1998/03620/problems_with_the_laryngeal_mask_airway_.11.aspx 10.1097/00004311-199803620-00011 9704277

[7] Michalek P, Donaldson W, Vobrubova E, Hakl M. Complications associated with the use of supraglottic airway devices in perioperative medicine. Biomed Res Int. 2015;2015:1–13. 10.1155/2015/746560 PMC4691459 26783527

[8] Yan Cling, Zhang Y, qi yuan, Chen Y, Qv Z, yang, Zuo Mzhang. Comparison of SaCoVLM™ video laryngeal mask-guided intubation and i-gel combined with flexible bronchoscopy-guided intubation in airway management during general anesthesia: a non-inferiority study. BMC Anesthesiol. 2022;22(1):302. 36138363 10.1186/s12871-022-01843-x PMC9494909

[9] Sharma M, Sharma B, Gupta M, Panday BC, Sahai C, Sood J. A randomized comparative study of three supraglottic airway devices for controlled ventilation in anesthetized patients. J Anaesthesiol Clin Pharmacol. 2022;38(2):221–7. 36171925 10.4103/joacp.JOACP_465_20 PMC9511860

[10] De Jong A, Molinari N, Terzi N, Mongardon N, Arnal JM, Guitton C, et al. Early identification of patients at risk for difficult intubation in the intensive care unit. Am J Respir Crit Care Med. 2013;187(8):832–9. 23348979 10.1164/rccm.201210-1851OC

[11] Sim J, Lewis M. The size of a pilot study for a clinical trial should be calculated in relation to considerations of precision and efficiency. J Clin Epidemiol. 2012;65(3):301–8. 22169081 10.1016/j.jclinepi.2011.07.011

[12] Karel Černý Vladimír. Herold Ivan Kozlík Pavel Šturma Jan C. Zásady bezpečné anesteziologické péče Pracovní skupina. 2009. https://www.csarim.cz/getmedia/790362a0-18c8-4553-97e1-1904e6d5ce28/zasady-bezpecne-anesteziologicke-pece-2009.pdf.aspx

[13] Keller C, Brimacombe JR, Keller K, Morris R. Comparison of four methods for assessing airway sealing pressure with the laryngeal mask airway in adult patients. Br J Anaesth. 1999;82(2):286–7. 10365012 10.1093/bja/82.2.286

[14] Shin HW, Yoo HN, Bae GE, Chang JC, Park MK, You HS, et al. Comparison of oropharyngeal leak pressure and clinical performance of LMA ProSeal™ and i-gel^®^ in adults: Meta-analysis and systematic review. J Int Med Res. 2016;44(3):405–18. 27009026 10.1177/0300060515607386 PMC5536706

[15] Lopez-Gil M, Brimacombe J, Keller C. A comparison of four methods for assessing oropharyngeal leak pressure with the laryngeal mask airway (LMA ^TM^) in paediatric patients. Pediatr Anesth. 2001;11(3):319–21. 10.1046/j.1460-9592.2001.00649.x 11359590

[16] Brain AIJ. The laryngeal mask—a new concept in airway management. Br J Anaesth. 1983;55(8):801–5. 6349667 10.1093/bja/55.8.801

[17] Chew EFF, Hashim NHM, Wang CY. Randomised comparison of the LMA Supreme™ with the I-Gel™ in spontaneously breathing anaesthetised adult patients. Anaesth Intensive Care. 2010;38(6):1018–22. 21226431 10.1177/0310057X1003800609

[18] Wong DT, Yang JJ, Jagannathan N. Brief review: the LMA Supreme™ supraglottic airway. Can J Anesthesia/Journal Canadien d’anesthésie. 2012;59(5):483–93. 10.1007/s12630-012-9673-0 22318376

[19] Teoh WHL, Lee KM, Suhitharan T, Yahaya Z, Teo MM, Sia ATH. Comparison of the LMA supreme vs the i-gel™ in paralysed patients undergoing gynaecological laparoscopic surgery with controlled ventilation*. Anaesthesia. 2010;65(12):1173–9. 20958278 10.1111/j.1365-2044.2010.06534.x

[20] Seet E, Rajeev S, Firoz T, Yousaf F, Wong J, Wong DT, et al. Safety and efficacy of laryngeal mask airway supreme versus laryngeal mask airway proseal: a randomized controlled trial. Eur J Anaesthesiol. 2010;27(7):602–7. 20540172 10.1097/eja.0b013e32833679e3

[21] Moser B, Keller C, Audigé L, Bruppacher HR. Oropharyngeal leak pressure of the LMA Protector™ vs the LMA Supreme™; a prospective, randomized, controlled clinical trial. Acta Anaesthesiol Scand. 2019;63(3):322–8. 30229857 10.1111/aas.13256

[22] Hwang J, Hong B, Kim YH, Lee WH, Jo Y, Youn S, et al. Comparison of laryngeal mask airway supremetm as non-inflatable cuff device and self-pressurized air-QTM in children. Medicine. 2019;98(10):e14746. 30855468 10.1097/MD.0000000000014746 PMC6417551

[23] Tan Y, Jiang J, Wang R. Contrast of oropharyngeal leak pressure and clinical performance of I-gel™ and LMA ProSeal™ in patients: A meta-analysis. PLoS ONE. 2022;17(12):e0278871. 36520843 10.1371/journal.pone.0278871 PMC9754199

[24] Kundal R, Puri K, Agrawal G, Singh R, Pandey M. A randomized controlled study to compare oropharyngeal leak pressure between I-gel™ and laryngeal mask airway supreme™ in children in lateral position under general anesthesia. Ain-Shams J Anesthesiology. 2023;15(1):17.

[25] Kim GW, Kim JY, Kim SJ, Moon YR, Park EJ, Park SY. Conditions for laryngeal mask airway placement in terms of oropharyngeal leak pressure: a comparison between blind insertion and laryngoscope-guided insertion. BMC Anesthesiol. 2019;19(1):4. 30611202 10.1186/s12871-018-0674-6 PMC6320569

[26] Singh A, Kaur J, Kaur S, Gupta KK. Safety and efficacy of LMA Supreme™ vs. LMA ProSeal™ for ambulatory surgeries in adult patients. Anaesth Pain Intensive Care. 2022;26(1):63–8.

[27] Katika SG, Madhuri SS, Murthy Sistla GK, Anmangi Subramanya KR, Comparison of i-gel and, lma supreme in patients undergoing elective surgeries under general anaesthesia. J Evid Based Med Healthc. 2017;4(4):159–62.

